# Contact-Lens Biosensors

**DOI:** 10.3390/s18082651

**Published:** 2018-08-13

**Authors:** Ryan Chang Tseng, Ching-Chuen Chen, Sheng-Min Hsu, Han-Sheng Chuang

**Affiliations:** 1 Department of Biomedical Engineering, National Cheng Kung University, Tainan City 701, Taiwan; tsengryan@gmail.com (R.C.T.); st86481234@gmail.com (C.-C.C.); 2 Department of Ophthalmology, National Cheng Kung University Hospital, Tainan City 704, Taiwan; shengmin@mail.ncku.edu.tw; 3 Medical Device Innovation Center, National Cheng Kung University, Tainan City 701, Taiwan

**Keywords:** biosensor, biomarker, contact lens, wearable device, tear

## Abstract

Rapid diagnosis and screening of diseases have become increasingly important in predictive and preventive medicine as they improve patient treatment strategies and reduce cost as well as burden on our healthcare system. In this regard, wearable devices are emerging as effective and reliable point-of-care diagnostics that can allow users to monitor their health at home. These wrist-worn, head-mounted, smart-textile, or smart-patches devices can offer valuable information on the conditions of patients as a non-invasive form of monitoring. However, they are significantly limited in monitoring physiological signals and biomechanics, and, mostly, rely on the physical attributes. Recently, developed wearable devices utilize body fluids, such as sweat, saliva, or skin interstitial fluid, and electrochemical interactions to allow continuous physiological condition and disease monitoring for users. Among them, tear fluid has been widely utilized in the investigation of ocular diseases, diabetes, and even cancers, because of its easy accessibility, lower complexity, and minimal invasiveness. By determining the concentration change of analytes within the tear fluid, it would be possible to identify disease progression and allow patient-oriented therapies. Considering the emerging trend of tear-based biosensing technology, this review article aims to focus on an overview of the tear fluid as a detection medium for certain diseases, such as ocular disorders, diabetes, and cancer. In addition, the rise and application of minimally invasive detection and monitoring via integrated contact lens biosensors will also be addressed, in regards to their practicality and current developmental progress.

## 1. Introduction

The rapid advancement of technology has enabled the realization of personalized medicine. According to the global demographics of health reported by the World Health Organization, 75% of the world population is defined as sub-health, but only 20% needed to be hospitalized. As the sub-health population has become more concerned about their quality of life, the demand for so called point-of-care (POC) diagnostics is rising [1]. Among POC products, wearable devices have drawn more public attention in the field of preventative and personalized medical care in recent years because of their convenience, high flexibility, and ability for long-term monitoring [2]. Wearable devices feature rapid screening, reduced assay costs with minimal sampling and efficient platforms for disease detection and monitoring.

The innovative progress in data processing power, high-bandwidth wireless communications, and the design of electronics and sensors has seen a surge in mainstream wearable healthcare devices that are lightweight, miniature, and effortlessly worn by users [3]. These wearable devices are capable of measuring the blood pressure, heart rate, blood oxygen levels, body temperature, and offer continuous monitoring to the users’ wellbeing. Well-known examples of such wearable devices are the Apple Watch, Samsung Gear series, Huawei wearable series, Garmin Vivo series, FitBit, and so forth [4,5,6,7,8]. 

**Figure 1:**
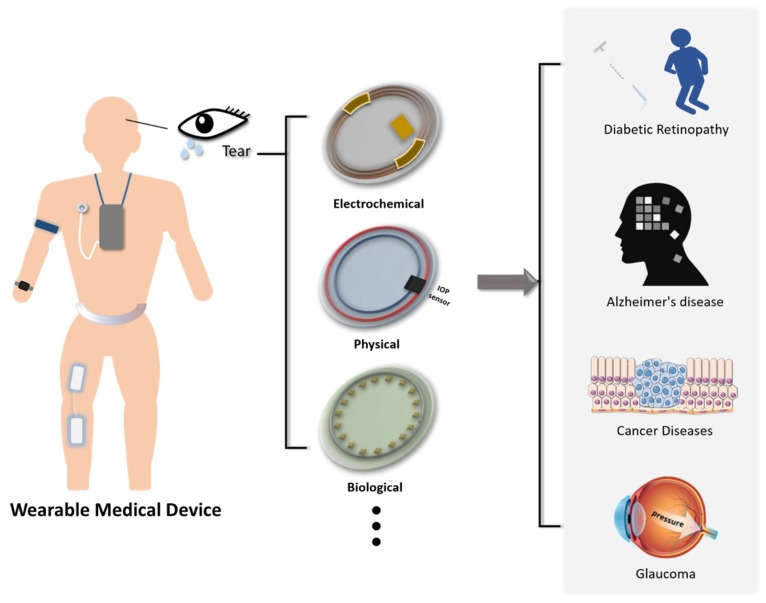
Overview of tear-based wearable medical devices.

Among these products, wrist-worn watches or smart-patches have received considerable attention from the public since they can track disease progression more frequently by detecting biochemical changes in sweat [9]. Additional investigation to establish correlations between blood and sweat remains necessary [10]. Nonetheless, sweat lacks protein biomarkers, thereby limiting its use in further disease detection [11]. Saliva and skin interstitial fluid (ISF) are also popular diagnostic samples in noninvasive wearable devices [12,13]. While saliva is typically sampled by intra-oral swabs, ISF can be collected by simply natural excretion. For saliva sensors, a novel device by placing a dental tattoo on a tooth to monitor the oral bacteria was developed by Manoor et al. [14]. However, their impedance sensor requires complicated and expensive instruments to retrieve data. Cross contamination of analytes and unfriendly environments in the mouth may compromise the accuracy as well [13]. For ISF sensors, detection of inherited metabolic diseases, organ failure, and drug efficacy were all exploited over the past decades. This technique even led to a commercial glucose monitoring device, GluoWatch Automatic Glucose Biographer Manufacturer (Cygnus, Inc., Redwood City, CA, USA). Unfortunately, skin irritation reported by patients led to subsequent withdrawal of the device from the market [12]. By contrast, tear fluid contains relatively rich proteins that have been identified and share similar constituents to that of blood [15]. This similarity is attributed to the plasma leakage across the blood-tear barrier and thus enables the development of biosensors that specifically target analytes within the tear fluid. Although blood testing remains second to none in the standard liquid biopsy, tear fluid can be collected in a non-invasive way. This feature makes tear fluid a promising sample in some preliminary clinical tests [16,17,18]. Followed by this concept, contact lenses have become an ideal platform for such kind of diagnosis as considering their irreplaceable popularity in our life (Figure 1). An emerging trend shows that researchers have begun to put more emphasis on the diagnostic potential of contact lenses with various lacrimal biomarkers. Up to now, contact lenses have been successfully integrated with sensors for continuous monitoring of diabetes and glaucoma [19]. The attempted development of contact lens biosensors gradually sparks commercial interests and initiates a brand new market. A recently eye-catching collaboration between Google and Novartis came up with a smart contact lens for diabetes management [20,21]. Ultimately, the integration of wearable devices with the Internet of Things and patient records will allow for more efficient implementation of treatment strategies.

Despite the promising future, contact lens biosensors remain in their infancy. Emerging challenges may come from long-term durability, robustness of driving mechanism, ease of use, and even the clinical perspectives. These factors may determine this new market to rise or fall in the end. In this review, we particularly focus on tear-based techniques with biomarkers for disease diagnoses. At last, their latest applications in contact lens biosensors will be explicitly addressed as well.

### 1.1. Essentials of Tear fluid

**Figure 2:**
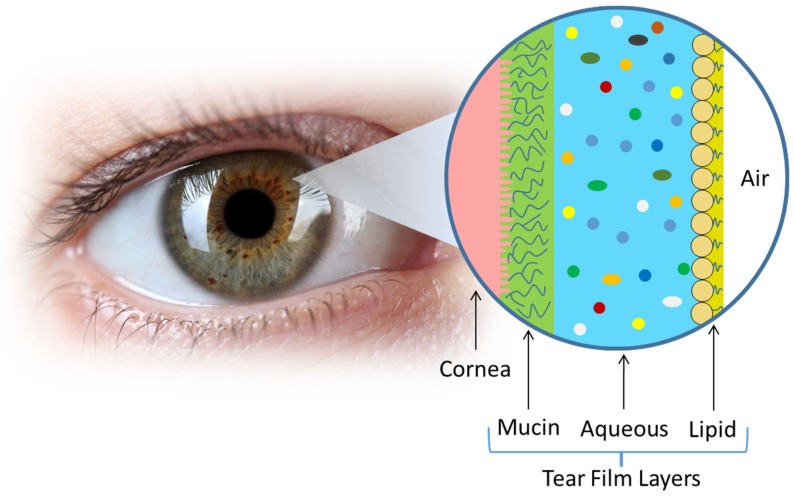
Schematic of the general configuration of human tear film.

The quest in search of alternative samples in place of blood for noninvasive diagnosis has been under intense investigation for decades [22,23]. However, none of them can shake the dominant role of blood to be a major standard sample fluid in clinical practices up to date. This long-established standard of procedure impedes the prevalence of other choices and sometimes results in reluctance of the humongous clinical community to adopt new measures. Despite the matter of fact, there remain hopes for other body fluids. In some cases, they possess unique merits in diagnosing specific diseases rather than blood. For example, bladder cancer is a urological disease resulting from complicated factors, such as smoking, family history, frequent bladder infections, and long exposure to certain chemicals. Urine is therefore an ideal sample fluid due to it direct contact with the morbid tissues. Among body fluids, tear (or lacrimal) fluid is a complex multilayered concoction of proteins, lipids, enzymes, and salts. As a result, a variety of biomarkers are present in the fluid and can be potentially used for disease screening [24]. In general, the tear film consists of three major layers (Figure 2 and Table 1) that functions as a lubricant and cleansing agent for the eye [25]. Notably, the tear fluid, in part, shares compositional similarities with the blood, because of plasma leakage across the blood-tear barrier [19]. Although the majority of components differ between the two body fluids, the plasma leakage allows examinations of the tear fluid in lieu of blood. Prior studies have shown that components, such as glucose, Na^+^, K^+^, and Cl^−^ ions, can be found in both the blood and tear fluid (Table 2). Nonetheless, tear fluid is proven to be a less complex body fluid as compared with serum or plasma because of the blood-tear barrier [26,27]. This strong filtering effect makes tear fluid contain significantly less proteins than its blood counterpart [11]. The reported number of proteins detected within the tear fluid is believed to be between 54 and 1543. The variation in the number of proteins can be highly dependent on the sampling method [28]. In this regard, there are three important tear types that can alter the composition of tears: (1) basal—the protective tear fluid produced during normal daily operation of the eye that covers the corneal surface; (2) reflex—secreted due to physical or chemical stimulation and to help remove irritants such as, foreign particles, vapors and gas, bright lights, or the action of coughing or yawning; and (3) psychic—induced by emotions (anger, pleasure, or pain), and contains higher concentrations of hormones [25].

Sampling methods can affect the composition of the tear fluid collected [31]. For example, Schirmer strips are likely to be in contact with the cornea; resulting in reflex tear secretion, alternatively microcapillary tubes can be used [32,33]. Although the use of these tubes is less invasive than Schirmer strips, however, the collection process remains time-consuming. Both of these methods are also limited to low volume tear collection, such that they become problematic when it comes to patients with dry eye disease. In addition, the tear collection is usually pointwise and discrete in time, which may be unsuitable to indicate the status of some diseases, such as diabetes, because the level of the biomarker may vary from time to time.

In addition to the problems from sampling methods, physiological stimulations are likely to alter the compositions of tear fluid. In particular, flow-dependent concentration effects, low sample volumes, and low concentrations of analytes within the fluid all require highly sensitive analysis techniques [34]. Another factor to be considered is the use of medication or supplements by patients (healthy or otherwise), can impact the compositions of tear fluid as well [35]. Despite all these difficulties mentioned above, past studies of lacrimal fluids have successfully identified various biomarkers used in the diagnosis and monitoring of disease and disorders. As such, many biosensors have been developed that utilize the tear fluid for the detection of disease biomarkers.

### 1.2. Why Contact Lenses?

**Figure 3:**
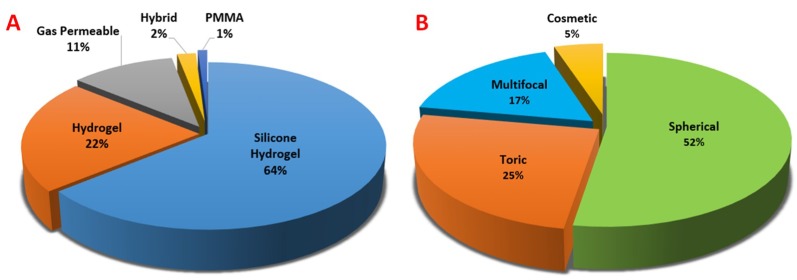
Contact lens use in 2017 by (**A**) materials class and (**B**) soft lens applications. Spherical lenses are, typically, used to correct myopia or hyperopia while toric lenses may help to correct astigmatism. Multifocal lenses can correct presbyopia and cosmetic lenses are designed to change the eye color with no particular medical purpose. Adapted with permission of Contact Lens Spectrum [49]. Copyright 2018, PentaVision LLC.

Contact lenses are the most popular wearable devices designed for vision correction, aesthetic, and therapeutic purposes all around the world. An estimated population of more than 45 million people in the United States rely on contact lenses daily [46]. Contact lenses are the only material allowed to be independently operated by users themselves and can provide physical contact with the human tissues for hours without serious irritation. The intimate relationship with eyes imparts contact lenses the possibility to develop as ideal carriers for long-term sensing. Blinking and tear secretion also allow for natural, fresh sample replenishment for reliable analyte collection throughout the day. Significant progress in the fabrication process and materials used has increased contact lens accessibility and appeal to consumers, making the cost for daily disposable lenses to be roughly US $1 [47,48]. Currently, the majority of contact lenses consist of soft materials based on proprietary polymer mixtures, while a few rigid contact lenses are still made of poly(methyl methacrylate) (PMMA) (Figure 3). They provide unique platforms for continuous monitoring of ocular status over a long period and can facilitate tear fluid sampling. Soft lenses, such as those made up of poly(2-hydroxyethyl methacrylate) (pHEMA), poly(vinyl alcohol) (PVA), polyacrylamide (PA), polyethylene terephthalate (PET), or polydimethylsiloxane (PDMS), are more commonly used in integrating sensing technology because of the prevalence of use, user comfort, and high oxygen permeability [19]. Moreover, contact lenses are portable in size and considered minimally invasive medical devices with the capability of integrating a variety of sensing techniques through surface or structural modifications. This feature of minimal invasion can prevent changes in the tear fluid proteomes and metabolite profile from external irritation. All above reasons make them ideal biosensing substrates among the modern wearable devices. By contrast, conventional sampling methods require the invasive acquisition of blood or serum for testing, which is considered inconvenient and painful accompanied by a great risk of infection. 

## 2. Tear Fluid Analysis

### 2.1. Biomarkers in Tear Fluid

Despite a relatively simple fluid, tear still contains a wide variety of proteins suitable for the detection of ocular and systemic disorders, such as dry eye syndrome (DES), ocular allergies, keratoconus (and keratopathy), trachoma, thyroid-associated orbitopathy, glaucoma, diabetic retinopathy (DR), systemic sclerosis, cystic fibrosis, cancer, multiple sclerosis, and Parkinson’s disease amongst others (Table 3). Numerous biomarkers have been correlated with specific diseases or disorders and can change the proteome profile of the tear fluid. By measuring the presence of certain proteins or metabolites from the liquid biopsy, their corresponding disease progression can be simply monitored and studied.

#### 2.1.1. Dry Eye Syndrome (DES)

A considerable number of studies on tear fluid have focused on the DES owing to the fact that it is a multifactorial disorder that can lead to ocular surface damage, irritation, visual disruption, and inflammation. This disorder is classified as evaporative dry eye or aqueous tear-deficient dry eye (ADDE). Medical practitioners are constantly facing difficulties in finding suitable treatment strategies because of the lack of adequate tools for monitoring and tracking patient responses. Present studies have analyzed cytokines, chemokines, growth factors, mucins, neuromediators, and lipids to identify protein profiles that can serve as target biomarkers for the DES. Aluru and colleagues [52] found correlations between several types of the DES and the down-regulation of lysozyme proline-rich protein 4. They hence suggested this protein a candidate biomarker for the DES. Another study reported by Zhou and colleagues [53] identified the up-regulation of α-enolase, α-1 acid glycoprotein 1, calgranulin A, calgranulin B, and calgizzarin, in addition to four down-regulated proteins, prolactin-inducible protein, lipocalin-1 (LCN-1), lactoferrin, and lysozyme in the DES patients. Despite many potential biomarkers under investigation, different research groups, up to now, have proposed other proteins relevant to the DES. For example, mucin (MUC)5AC was found to decline, but malate dehydrogenase 2 (MDH2) showed an increased activity in the DES patients [54,55]. Surprisingly, neuromediators, such as calcitonin gene-related peptide (CGRP), neuropeptide Y (NPY), vasointestinal peptide, and nerve growth factor (NGF), have been revealed as potential biomarkers for the DES as they have been linked in clinical studies [56]. Lambiase et al. [56] discovered that the DES-affected patients showed increased levels of NGF, and reduced levels of CGRP and NPY, with respect to DES severity. Cytokines and chemokines have also been identified as potential targets for the DES diagnosis as considering the inflammatory nature of the disorder. Several inflammatory proteins, such as interleukin-1 (IL-1), IL-6, TNF-α, metalloproteinase-9 (MMP-9), IL-17A, and IL-1RA showed elevated expression in the tears of the DES patients [17,57,58,59,60,61]. Accordingly, a POC device was developed and commercialized based on MMP-9, which could indicate DES severity [18,62]. In addition to proteomics, lipidomics and metabolomics have also been attempted by many researchers recently in order to exploit more promising biomarkers for precise diagnosis of the DES [63,64].

#### 2.1.2. Diabetic Retinopathy (DR)

Multiple potential biomarkers in tear fluid have also been identified for systemic diseases, such as diabetes, cystic fibrosis, and sclerosis. Among the diseases, diabetes mellitus is a chronic metabolic disease that affects over 400 million people, globally. The total expenditure of diabetes treatments exceeds US $670 billion and is expected to escalate to US $800 billion by 2040 [65]. Diabetes is known as the major cause of DR. DR is a common and severe ocular disease that can eventually lead to complications such as vitreous hemorrhaging, retinal detachment, glaucoma, and blindness [66,67]. To better understand DR, Csosz and colleagues [68] successfully identified several potential biomarkers, including LCN-1, lactotransferrin, lysozyme C, lacritin, and lipophilin A. Kim and colleagues [69] then confirmed DR patients through the analysis of LCN-1, heat-shock protein 27, and beta-2 microglobulin expressions. Later, Costagliola and his colleagues [70] reported that DR patients expressed increased levels of tumor necrosis factor alpha (TNF-α), verifying the possible link between TNF-α and DR severity. Furthermore, the concentration of NGF within the tears of patients with proliferative DR was found at increased levels compared to those of non-proliferative DR and non-diabetic patients, implying the potential use in determining patient affliction [71]. Conversely, the concentrations of endothelin and neuron-specific enolase in non-proliferative DR patients were found to escalate [24]. Although the relationship between the proteins and DR seems to be quite diverse from one to another, they may be able to form a combination of various biomarkers to assist in more accurate detection of the status of DR in patients. The identifications of the overall biomarkers provide new avenues to develop personalized diagnostic and screening tools depending on needs. However, the use of specialized equipment for analysis will need to be addressed before the utility of these biomarkers are realized in POC applications.

#### 2.1.3. Cancers

More and more evidence has shown that cancer biomarkers can be found within the tear fluid in the recent years. Such as the study reported by Evans et al. [72], where the levels of lacryglobin in tears were correlated with breast cancer in patients. In their groundbreaking study, the group tested tear samples from patients with different cancers, and reported that lacryglobin was found in tear fluid of those affected with colon, prostate, breast, lung and ovarian cancers. Additionally, control subjects displaying the presence of lacryglobin had ancestors with breast and prostate cancer. This study demonstrated that tear fluid biomarkers can enable the non-invasive detection of cancer. Later Lebrecht et al. [73,74] demonstrated that it was possible to differentiate patients with breast cancer from the control subjects. In addition, the Bohm group [75] identified several breast carcinoma biomarkers in the tear fluid, such as complement C1q subcomponent subunit C or protein S100A8, and metabolites aldehyde dehydrogenase 3A or triosephosphate isomerase. They confirmed the expression of these biomarkers were altered in cancer patients, in comparison with the control subjects. Nonetheless, only a few studies have put effort into investigating possible biomarkers for cancers within the tear fluid. The fact is likely attributed to the sheer number of different types of cancers, such that discovery has been hampered. In addition, many cancers share similar biomarkers resulting in difficulty in pinpointing specific types of cancers via this method unless a multiplexed detection system is employed. However, it is worth noting that being able to gauge the susceptibility of an individual to cancer and early detection, as demonstrated in Evans’ study [72], can aid physicians in providing better care for patients.

#### 2.1.4. Cystic Fibrosis and Others

Cystic fibrosis is a genetic disorder that affects the exocrine glands that causes the abnormal accumulation of mucus within the lungs, pancreas, intestines, and sweat glands [76]. The onset of this disorder can lead to lung infections and digestive problems that are life-threatening. In addition, cystic fibrosis can also cause dry eye symptoms in patients, as it affects the secretion of epithelial cells [77]. It was found by Mrugacz and his colleagues [77] that IL-8 and IFN-γ expression correlated with inflammation of the ocular surface, as well as cystic fibrosis pathology. As we can expect, more culprits were associated with this disorder as more research studies were conducted. For example, macrophage inflammatory protein 1 alpha (MIP-1α) and MIP-1β were later confirmed to play important roles in the inflammatory responses in patients affected with cystic fibrosis [78,79]. Lately, researchers have attempted to turn their attention to neurological disorders, such as multiple sclerosis and Parkinson’s disease. Surprisingly, the biomarkers of neurodegenerative diseases, α-1-antichymotrypsin and TNF-α, have been identified in tear fluid [80,81]. For a general overview of the abovementioned biomarkers and disease, Table 3 provides a comprehensive list of potential tear fluid biomarkers that can be used in the detection and monitoring of ocular and systemic diseases.

## 3. Contact-Lens Biosensors

**Figure 4:**
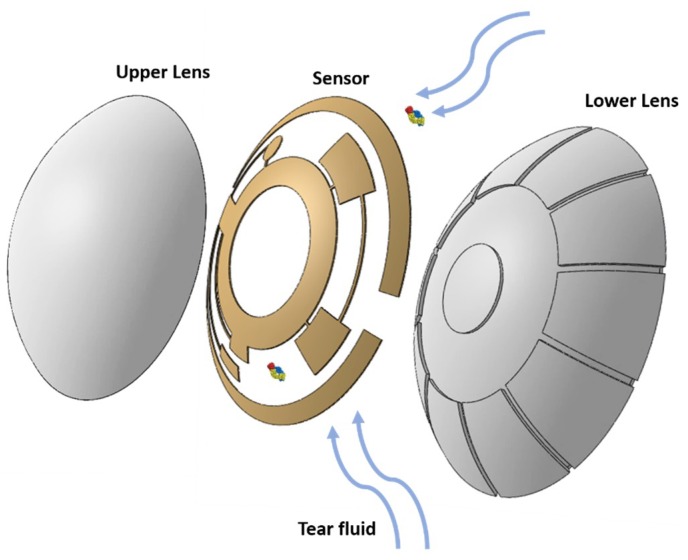
Conceptual illustration of a contact lens biosensor.

The identification of potential biomarkers for the early detection of diseases and disorders is still ongoing. Currently known biomarkers are being tested in biosensors for POC applications, which range from on-chip sensors to functional integrated sensors, such as contact lenses. Since a large market has taken shape in diabetes management, research and development of contact lens sensors are predominantly focused on the glucose-related field. As the sensing technology advances, the range of diseases that can be monitored and detected via contact lens biosensors will increase. Continuous monitoring may not be required for many diseases, and contact lens biosensors can also serve as a one-time use diagnostic tool. In this case, contact lenses will naturally accumulate tear components during wear and can be analyzed after wear. By integrating the detection of specific biomarkers, such as in cancer, or dry eye, it would be possible to identify the presence and progression of certain diseases (Figure 4). This section lays emphasis on the major emerging methods of detection, such as fluorescent, holographic, colorimetric, and electrochemical, based on contact lenses. A performance comparison of different types of contact lens biosensors is detailed in Table 4. Cutting-edge developments that may have potential for integration will be discussed in the following as well. 

### 3.1. Fluorescence-Based Sensing

**Figure 5:**
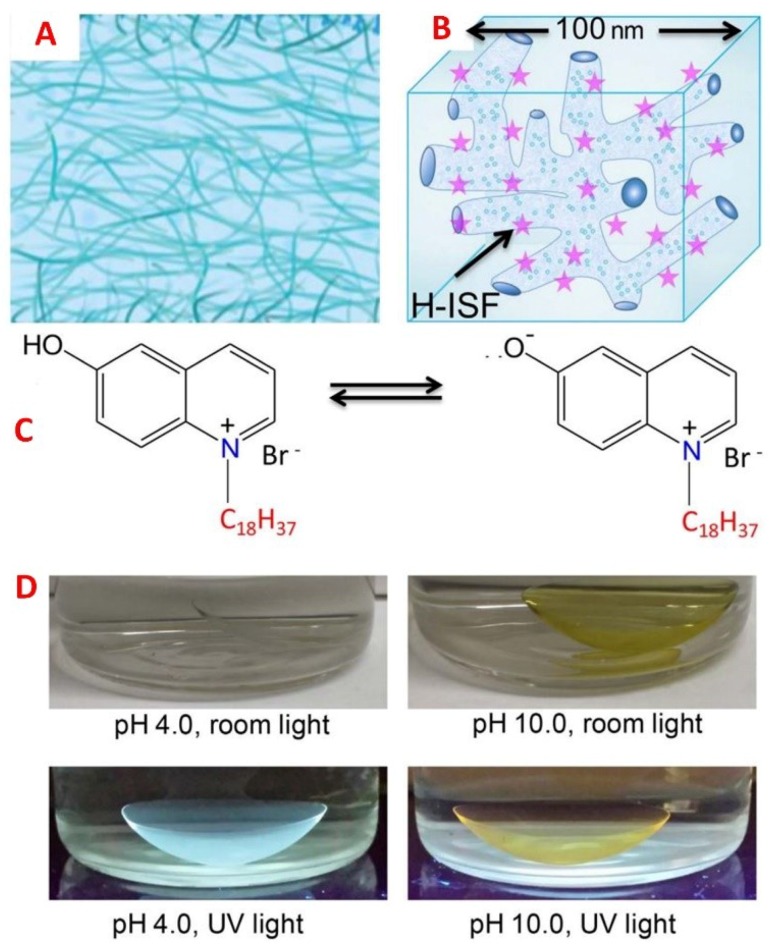
(**A**) Schematic of the non-silicone hydrogel contact lens showing cross-linked polymer strands that are spatially homogenous as compared to (**B**) the silicone hydrogel lenses with hydrophilic and hydrophobic regions that facilitate the binding of hydrophobic ion-sensitive fluorophores. (**C**) The neutral and anionic equilibrium of pH probe, 6HQ-C18. (**D**) Images of a contact lens labelled with 6HQ-C18 at pH 4.0 and 10.0 under ambient or UV light. Reprinted and adapted with permission [88]. Copyright 2018, Elsevier.

Fluorescence-based sensing has been utilized in a plethora of applications owing to its versatility, sensitivity, and specificity. The basis of fluorescence is the absorption of electromagnetic radiation of a specific wavelength by an excitable fluorophore and the subsequent emission of photons with longer wavelength. The released photons can then be differentiated from background noise with filtering techniques, making this detection technique highly sensitive. The excited and emitted photonic wavelengths are dependent on the chemical structure of the fluorophore, which allows customizable and highly specific fluorescence sensing. In relation to contact lens applications, a biosensor for glucose detection through immersion in tear fluid was developed early on by March et al. [87] with tetramethylrhodamine isothicyanate concanavalin A (TRITC-Con A) and fluorescein isothiocyanate dextran (FITC-dextran) encapsulated within hydrogel spheres that were embedded and immobilized in polymerized Nelfilcon A (PVA-based) within a contact lens mold. As glucose diffuses into the spheres, the FITC-dextran molecules are shifted away from TRITC-Con A, which results in a decreased Forster Resonance Energy Transfer (FRET) and the increase of fluorescence intensity. This biosensor was able to track the concentration of blood glucose of patients over three hours. However, a delay of 15 min between the blood glucose concentration and readout from the sensor was found. The biosensor was compatible with a hand-held photofluorometer, which obtained the green fluorescent readout signal. It was proposed that the photofluorometer could also be used in conjunction with an insulin pump (or similar) to be used in the management of diabetes. In another study, a contact lens biosensor, using pHEMA or PDMS, embedded with organic dyes encapsulated within silica nanoparticles was proposed. This biosensor was able to detect glucose in the range of 0.5 and 5 mM. The use of silica maintained the capsule shell integrity and prevented premature leakage. Similarly, Badugu and his colleagues [44,45,88,89,90] developed boronic acid-based probes for tear glucose detection and then successfully integrated them into commercially-available contact lenses. Their study demonstrated that the probes could readily detect tear glucose changes within the range from 50 μM to 100 mM for diabetics. The contact lens biosensor had a response time of 10 min, such that it allowed continuous and non-invasive monitoring of physiological glucose and reducing the need for invasive blood sampling. More recently, the Lakowicz group [88] attempted to fabricate a contact lens platform to evaluate the ion concentrations in tear fluid via fluorescence. The use of commercially available silicone hydrogel contact lenses enabled the binding of hydrophobic ion-sensitive fluorophores. Notably, the silicone-based contact lenses can facilitate the rapid transport of both oxygen and tear fluid, hence they are feasible for long-term use [91]. Other than proteins, Badugu et al. [88] demonstrated that functionalizing silicone contact lenses with fluorescent probes could even detect changes in chloride ion concentration and pH (Figure 5). The proof of concept therefore paved a way for more probes for use in the detection of sodium, potassium, calcium, and magnesium ion concentrations. The same research group hopes to create a multiplexed platform by functionalizing various regions of a contact lens to measure the concentrations of tear electrolytes. In general, the development of water-soluble fluorescent probes for biosensing offers various advantages such as high specificity, versatility, and potential for easy analysis via handheld readers/detectors [16,87]. By properly incorporating immunofluorescent assays into contact lenses, it would be possible to detect a wide variety of biomarkers, such that diseases can be diagnosed simply through non-invasive liquid biopsy alone.

### 3.2. Photonic-Based Sensing Structures

#### 3.2.1. One-Dimensional Photonic Crystal: Holographic Gratings

Photonic crystal (PC) are formed by periodically-ordered structures and can be classified into one dimension, two dimensions, and three dimensions according to its optical orientation. When a PC structure is illuminated by white light, light will then be diffracted according to Bragg’s Law:

(1) mλ=2ndsinθ

where *m* is the diffraction order, *λ* is the wavelength of incident light, *n* is the refractive index of the matrix, *d* is the spacing of the PC plane, and *θ* is the received angle of the photodetector. Accordingly, the diffracted light will change color as its wavelength shifts owing to spacing or refractive index changes. Based on this principle, holographic gratings (1D PC) are frequently used as a measure to show minute changes on the PC plane. By modifying the grating surfaces, a holographic sensor is able to change its optical characteristics when the target analytes are present. Since the sensing usually accompanied with color changes, PC have become a popular way in visible medical devices. Such holographic methods were utilized as photonic structures to serve as sensors in the quantification of glucose in tear fluid [92]. This method used a multilayered and periodic structure, which is then functionalized with compounds, such as derivatives of boronic acid [93]. The functionalized surface was then able to covalently capture carbohydrates (i.e., glucose). An example of this type of device was well demonstrated by Yetisen and colleagues [94]. Their sensor comprised glucose-binding 3-(acrylamido) phenylboronic acid (3-APB) which was functionalized on a PA-based hydrogel substrate. The reversible binding between glucose and 3-APB resulted in the swelling of the hydrogel sensor that led to the red-shift of the diffracted light. Holographic sensors offer various advantages: (1) no need for dyes or fluorophores to operate, eliminating photobleaching effects on the sensors; and (2) the potential integration with readily accessible technology, such as smartphone cameras, owing to the near-infrared capability.

**Figure 6:**
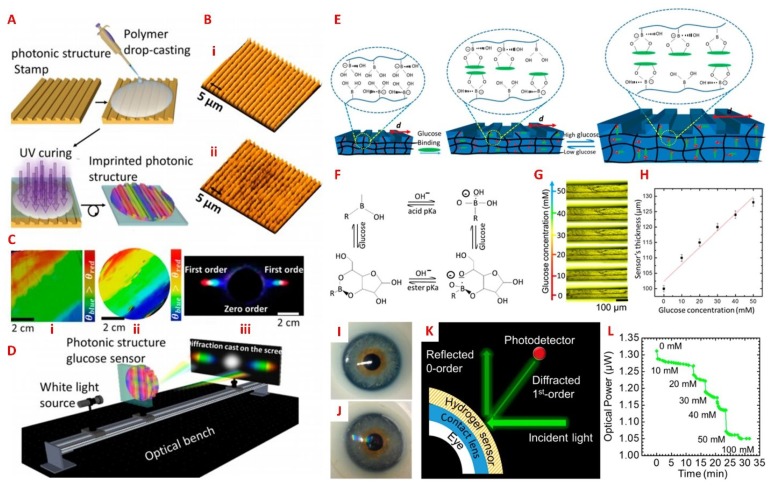
(**A**) Schematic of the fabrication process of a one-dimensional hydrogel glucose sensor. The replica photonic structures are produced through drop-casting and UV-curing of monomer solutions on a master stamp. (**B**) Images of the (i) photonic structure stamp master and (ii) the imprinted hydrogel structure. (**C**) Images of (i) the original grating; (ii) the imprinted hydrogel sensor; and (iii) the transmitted diffraction pattern for a white light source by the sensor, using the setup in (**D**). (**E**) The sensing principle of the PA-based photonic hydrogel sensor, where it is functionalized with 3-APB for detecting glucose. The reversible swelling of the photonic hydrogel sensor alters the refractive index, and shifts the wavelength of diffracted light. (**F**) The equilibrium between glucose and boronic acid probes. (**G**) Optical images of the sensor, and (**H**) the associated change in cross-sectional thickness at various glucose concentrations. (**I**) Image of a commercial contact lens and (**J**) glucose sensor integrated contact lens on an artificial eye model. (**K**) Schematic of the measurement setup for measuring (**L**) the reflected first-order diffraction at various glucose concentrations. Reprinted and adapted with permission from [82]. Copyright 2018, American Chemical Society.

With regards to contact lens biosensors, a hydrogel matrix consisting of acrylamide, 3-APB, and *N*,*N*′-methylenebisacrylamide (bis-AA) were studied. The hydrogel film was photopolymerized and embedded with silver bromide along with a dye to create a polymer film. The holographic film was then incorporated into a contact lens after treatment with PVA contact lens formulation [95]. The result was a contact lens with holographic diffraction gratings that can diffract light. When glucose entered the hydrogel it is bound to the cis-diol groups of 3-APB, causing the matrix to expand and alter the interparticle spacing of the nanoparticles. This preliminary study demonstrated that the glucose-binding was reversible and that the biosensor was able to track blood glucose levels via tear fluid. However, further investigation is required to fully characterize the contact lens biosensor. A main concern in the use of boronic acid derivatives with tear fluid is that the interaction and binding of other carbohydrates or hydroxyl acids, such as lactate, with comparable concentration to glucose can interfere with the measurements. Attempts to remedy the interference included optimizing the 3-APB mole fraction (approximately 0.2), which increased the sensitivity to glucose [96]. It was found that up to 20 mol % improved the Bragg shift, but over this value increased the hydrophobicity of the hydrogel. This finding thereby limited the matrix swell and reduced overall shift [92]. The study has demonstrated that this sensor has the potential for use as an ophthalmic glucose sensor. Recently, holographic structures were utilized within contact lenses to produce a low-cost and reusable glucose monitoring system (Figure 6) [82]. The optical glucose sensor was fabricated on a photonic structure with a periodicity of 1.6 µm. The structure was imprinted on a phenylboronic acid-functionalized hydrogel (PA-based) film, and was ultimately attached to a commercial contact lens. The wearable contact lens sensor was designed for use, in conjunction with smartphone technology for readouts, at POC settings. The volumetric change due to the binding of glucose alters the periodicity of the photonic structure, which changes the diffraction angle, and the subsequent diffracted wavelength of light. The fabricated contact lens sensor was demonstrated to have a response time of 3 s and a saturation period of 4 min with high sensitivity for glucose concentrations up to 50 mM. One of the issues that can affect its practical use is glaucoma. The change in intraocular pressure may also affect the readout produced by the photonic-based sensor because of disturbances in the periodic structure.

Holographic sensor integration with contact lenses offers a unique label-free solution to the detection of biomarkers. The versatility of functionalization for targeting specific analytes such as ions or proteins can be instrumental in the development of contact lens biosensors. Additionally, the use of diffraction gratings means that it is possible to pattern a variety of materials and diffracted wavelengths, while dictating the angle of diffraction.

#### 3.2.2. Multi-Dimensional Photonic Crystal: Colloidal Crystal Arrays (CCA)

In addition to the one-dimensional PC introduced previously, two- and three-dimensional PCs are tremendously developed for biosensing purposes as well. Photonic crystal array sensors are 3D PCs consisting of nano-sized particles immobilized within polymer matrices. These PCs are formed from orderly-stacked crystalline nanospheres, such as polystyrene or silica, and have been used in biosensors for glucose in tear fluid. The construction of CCA involves the self-assembly of monodispersed particles by taking advantage of the evaporation of the colloidal solutions. Alexeev et al. [97] were one of the first to develop photonic crystals in glucose sensing applications. Their sensor consisted of polystyrene colloids embedded within an PA-(bis-AA)-poly(ethylene glycol) matrix, and functionalized with boronic acid derivatives (4-acetamido-3-fluorophenylboronic acid and 3-fluoro-4-*N*-methylcarboxamide phenylboronic acid), which permitted the sensing of glucose at physiological pH. The measurement mechanism involves glucose binding to boronic acid and forming additional linkages within the polymer matrix, which results in the overall shrinkage and modification of the lattice spacing. The shrinkage of the matrix is proportional to the glucose concentration in the tear fluid and results in a blue shift of the diffracted light. In another study from the same research group [98], the sensitivity and response time of the CCA sensors were further improved for the detection of glucose concentrations in blood (5 mM) and tear fluid (0.15 mM). The optimal response times for blood and tear fluid measurements could respectively reach 90 s and 300 s at physiological pH and temperature. For the tear fluid, a blue shift of 11 nm was observed with a detection limit of 1 μM in synthetic tear fluid.

**Figure 7:**
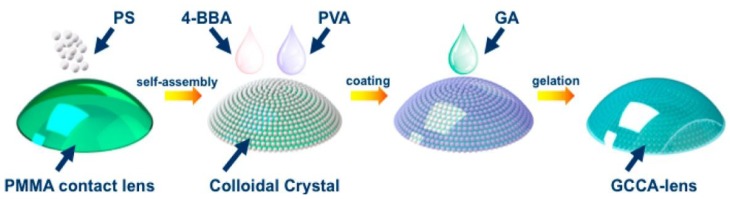
Preparation of glucose-detected gelated CCA-lens (GCCA-lens). The polystyrene particles self-assembled on the RGP contact lens and then a solution of 4-boronobenzaldehyde-modified poly(vinyl alcohol) (4-BBA-PVA) was coated on the colloidal crystal to form a gel. Finally, the gel was cross-linked by the addition of glutaraldehyde (GA). Reproduced with permission [84]. Copyright 2017, MDPI AG.

Lately, Zhang et al. [83] have demonstrated the use of colloidal array photonic crystal encapsulated within an acrylamide hydrogel for continuous glucose monitoring. Firstly, a suspension of polystyrene colloids was mixed with acrylamide monomers and polymerized. The PA hydrogel matrix was then partially hydrolyzed to generate carboxylate sites and converted to phenylboronic acid (PBA). The final volume of the hydrogel was established by the addition of PVA, which binds to the immobilized PBA sites, forming cross-links and causing the overall volume to shrink. The mechanism of glucose sensing involves the reversible formation of PBA-glucose complexes, causing the expansion of the hydrogel volume and increasing the wavelength of reflected light. Hence, the wavelength of reflected light is proportional to the hydrogel volume and glucose concentration. In this study, 0 to 50 mM of glucose in a buffered solution was used to evaluate the response of the material. A positive linear correlation between glucose concentration and the wavelength of reflected light (R^2^ = 0.998) was observed. Ruan et al. [84] further advanced this sensing technique and applied it directly to rigid gas permeable (RGP) contact lenses (Figure 7). They successfully modified commercially-available contact lenses to sense glucose by forming CCA on the surface and immobilizing the CCA layer with a glucose-responsive hydrogel. The study eventually made a sensor that was able to detect and differentiate glucose in buffered solution ranging from 0 to 50 Mm. Visible blue shifts from 0 to 3 mM (a wavelength shift from 525 to 468 nm) and red shifts were measured on this biosensor when the glucose concentration exceeded 10 mM (up to 567 nm at 50 mM). As the physiologically-relevant glucose concentration fell within the range between 0.1 to 0.6 mM, the two-way shifts can be considered negligible. In further testing with simulated tear fluid, the CCA sensor displayed a 10 nm diffraction wavelength shift from 0 mM to 0.6 mM with a relatively linear correlation. Although the sensitivity of the sensor within tear fluid was about one-third lower than buffered solution, the fabricated sensor was able to demonstrate that detection of glucose concentrations within physiological ranges was possible, and further improvements could lead to practical use.

CCAs formed from microgel spheres in glucose detection were also reported by Wu et al. [99], where glucose-sensitive poly(styrene-*co*-acrylamide-*co*-3-acrylamidophenylboronic acid) self-assembling spheres were embedded within a poly(acrylamide-*co*-2-(dimethylamino)ethyl acrylate) hydrogel matrix. The CCA-embedded hydrogel responded to various glucose concentrations by swelling, with a maximum swelling ratio of 2.02 at a concentration of 300 mg/dL, and could fully recover after the dissociation of glucose. The detection of glucose was performed using reflection spectra at 1722 nm (near-infrared), and it was demonstrated that the CCA-hydrogel was highly stable, even after five months of storage at room temperature. In addition, interference from non-sugar constituents in tears was found to have minimal impact on the reflection spectra with relative errors less than 3%. The results of the study revealed that the detection limit of the CCA-embedded hydrogel was 6.1 μg/dL, while physiologically relevant glucose concentration of 7.5 mg/dL could be determined within 22 s and reaching a maximum in 2 min. Shorter response times were achieved with higher glucose concentrations. The characteristics mentioned above demonstrated the potential of this material for a POC device, particularly incorporation within contact lenses. Although most materials presented here focus on boronic acid derivatives and glucose sensing, it may be possible to functionalize these CCA-embedded hydrogels with biomarker-specific antibodies for the detection of other diseases.

**Figure 8:**
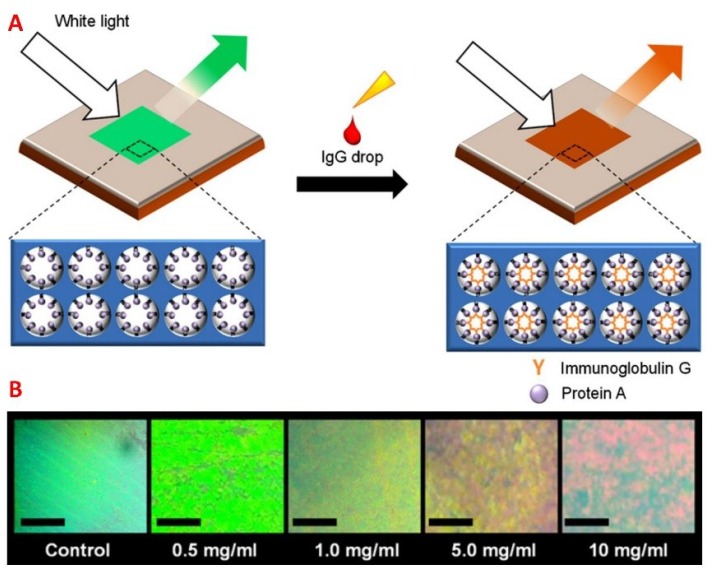
(**A**) Mechanism of the inverse-opal biosensor. The addition of IgG antibodies binding to the sensor causes a change to the refractive index of the matrix. (**B**) The change in color from green to red with increasing antibody concentrations observed under an optical microscopy. The scale bars are 0.5 mm. Reprinted and adapted with permission [100]. Copyright 2013, Elsevier.

Inverse-opal photonic crystals produced from CCAs have also been investigated for use in biosensing. Choi and colleagues [100] came up with a nanoporous photonic crystal structure within a hydrogel for use in the detection of immunoglobulin G (IgG) antibodies (Figure 8). The fabrication of their device involved the self-assembly of silica nanoparticles followed by immersion within a poly(ethylene glycol) diacrylate matrix and UV-curing. The silica nanoparticles were then etched away to reveal the nanoporous structure within the polymer matrix. The nanoporous structure was functionalized with Protein A that bound IgG antibodies and was able to effectively distinguish various concentrations of IgG antibodies. As the concentration of antibodies increased, the reflected wavelength red shifted to a unique peak at each concentration as a result of a change in the effective refractive index. The results of this study demonstrated that inverse-opal structures have the potential to be used in the detection of other analytes, and could be integrated within contact lenses to form wearable biosensors. However, the sensitivity of this type of sensor will need to be improved in order to detect trace amounts of biomarkers within tear fluid.

### 3.3. Electrochemical Sensing

**Figure 9:**
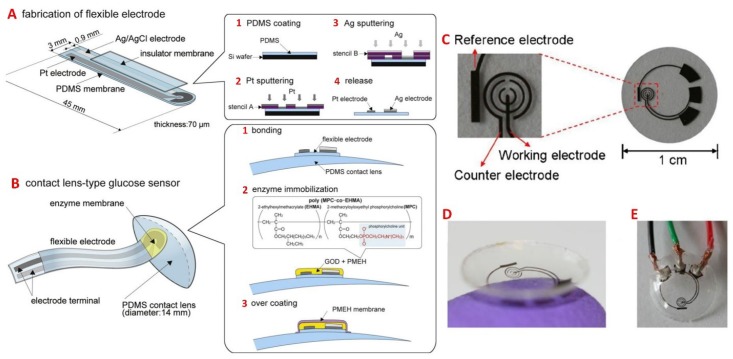
Fabrication process for a contact lens biosensor: (**A**) flexible electrodes, consisting of a 200 nm Pt working electrode and a 300 nm Ag/AgCl counter/reference electrode, were formed onto a 70-µm thick PDMS film. (**B**) The electrodes were then bonded onto a PDMS lens and coated with GOD as well as PMEH. Reproduced with permission [86]. Copyright 2011, Elsevier. (**C**) The circuitry used in the detection of glucose with reference, working, and counter electrodes. (**D**) Integration of the circuitry onto a contact lens and (**E**) the testing setup used. Adapted and reproduced with permission [39]. Copyright 2011, Elsevier.

Electrochemical sensors for bioanalyte detection have been well-researched over past decades. Various fabrication techniques, especially those used in the semiconductor industry, are commonly employed to construct biosensors [101]. In the case of glucose detection, the sensors utilize enzymatic action that allow for highly selective reactions to occur for electrochemical sensing. Generally, glucose oxidase (GOD) converts glucose to gluconolactone and hydrogen peroxide, which further dissociates into oxygen, hydrogen ions and electrons. The three-electrode system of the sensor utilizes the electrons produced to quantify the concentration of glucose in the fluid. These chip-based electrochemical sensors have also been integrated into contact lenses for the determination of glucose concentration in artificial tear fluid [39]. In this study, the sensor circuitry was fabricated using a photoresist (AZ4620) layer to pattern and deposit Ti/Pd/Pt onto a PET wafer. The resist layer was dissolved in acetone and the wafer was heat-molded into a contact lens, with the electrodes on the convex surface. Pre-treatment of the sensor required the GOD to be immobilized within a titania sol-gel film and applied to the sensing electrodes prior to testing of samples. The developed contact lens sensor had a detection limit of 0.01 mM glucose, approximately one-tenth of glucose concentration in human tears [15]. Similarly, Chu et al. [86] developed a contact lens biosensor for the in situ monitoring of tear glucose using PDMS as the contact lens material and a thin PDMS film as a base for the flexible hydrogen peroxide electrode (with Pt and Ag/AgCl as the working and reference/counter electrodes, respectively) (Figure 9). Initial in vitro testing confirmed that the biosensor was able to detect glucose concentration in the ranges of 0.03 to 5.0 mM, which covered the normal tear glucose levels in humans (0.14 mM). Subsequently, the in situ performance of the fabricated contact lens biosensor was investigated via introduction to the eyeball of a rabbit model and corroborated with conventional glucose blood tests. The glucose levels within the blood and tears were spiked by the oral intake of glucose solution by the rabbit, and it was demonstrated the biosensor was able to detect a change in the tear glucose levels with a delay of 10 min and peaked after 55 min.

**Figure 10:**
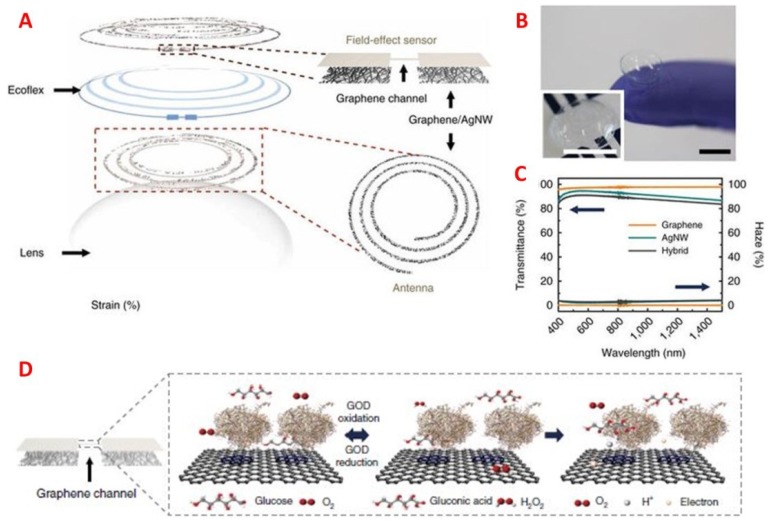
(**A**) Schematic of the wearable contact lens biosensor, integrating glucose, and intraocular pressure sensing. (**B**) Image of the fabricated contact lens sensor. The scale bar is 1 cm. (**C**) Optical transmittance and haze of graphene, silver nanowire film, and a hybrid structure. (**D**) Glucose detection with GOD-pyrene functionalized graphene. Reproduced and adapted with permission [102]. Copyright 2017, Springer Nature.

More recently, Kim et al. [102] developed graphene-based sensing for the simultaneous detection and monitoring of glucose and intraocular pressure without interference from the other (Figure 10). In their study, a biosensor consisting of graphene and silver nanowires were incorporated into contact lenses and detected glucose concentration via GOD-conjugated field-effect transistor sensor. The use of resistance, inductance, and capacitance (RLC) circuit enabled the measurements of molecular binding (R) and structural changes in the device (L-C) for glucose and intraocular pressure, respectively. This study demonstrated that pyrene-binding biomarker receivers onto graphene can be tuned for various analytes for the future development of a multiplexed contact lens sensor for identifying and evaluating disease via ocular diagnostics.

Biosensors targeting the lactate within tear fluid have also been developed using similar principles for glucose detection, where l-lactate is converted to pyruvate and hydrogen peroxide by lactate oxidase (LOD). In addition to developing glucose biosensors, the Parviz group [42] utilized the same fabrication process to develop a lactate-monitoring contact lens biosensor. The LOD was covalently immobilized at the sensing electrodes and it was found that the in vitro detection limit for this biosensor was 50 μM (approximately one-twentieth of the l-lactate found in the tear fluid) with an average response time of 35 s. One of the issues discovered was the interference of ascorbic acid on the detected current; although no interference was found with glucose or urea. To compensate for the interfering signal a dual sensor was utilized one for the detection of lactate and the other for ascorbic acid. In addition, the LOD biosensor was able to maintain its activity and current response after intermittent sampling under physiological conditions over a 24-h period. The ability of the biosensor to retain its enzymatic activity is important for practical applications that require packaging and transportation after fabrication; although further investigation into the storage conditions is required. Electrochemical-based sensing systems are appropriately suited as tear fluid diagnostics via the integration into contact lenses because they are highly selective, and provide rapid readouts. The new type of integration enables the detection and monitoring of low-concentration bioanalytes with a POC device.

## 4. Future Prospects

### 4.1. Challenges of Current Methods and Devices 

The use of boronic acid-based detection of glucose has been fundamental in developing colorimetric and fluorescent glucose detecting contact lens sensors that respond well to millimolar concentrations of glucose. However the sensitivity of these have been found to decrease after incorporation into contact lenses [44]. An issue with fluorescence-based detection systems is the choice of the fluorophore as photochemical stability is important for long periods of time in continuous monitoring cases, and many fluorophores display limited solubility in aqueous media. Additionally, lactate and other saccharides can be a major interfering species in boronic acid sensors as concentrations for lactate range from 1 to 5 mM in the tear fluid [103]. These issues affect both fluorescent and holographic sensors that utilize the boronic acid binding to glucose for detection. Hence, further investigation into increasing the sensitivity and selectivity of boronic acid-based sensors is required before use in clinical settings.

Biosensors that utilize enzymatic reactions, such as GOD and LOD, to produce electrochemical signal usually face issues with sensing stability. Although enzyme-based sensors show high sensitivity, selectivity, efficiency, and low cost, their downfall is the short lifespan enzymes have due to degradation [104]. This drawback limits enzymatic biosensors to be used within their short effective periods to ensure accuracy. Operating conditions, such as temperature, humidity, and pH can limit the general use of these enzymatic biosensors as well. In addition, contact lenses need to be sterilized, via autoclave according to regulations. Unfortunately, the sterilization process is likely to denature the essential enzymes. There are also issues related to effective immobilization of the enzymes, and the unwanted reaction of H_2_O_2_ and redox-active species, such as ascorbic acid that can interfere with the signal. Moreover, electrochemical sensors require external power to drive the reactions, which is problematic in developing contact lenses for long observation periods.

One major challenge to using contact lens biosensors is repeatability/stability. Obviously, this ability heavily depends on a well-established calibration for each device. The tedious procedure is potentially necessary for clinical environments due to background interferences. Big data acquired from clinical trials can certainly provide valuable information for such calibration purpose. However, a large number of patients needed for the database and expected years of work can also pose a challenging task to researchers who are interested. Other considerations of the adverse effects raise from continuously wearing contact lens, which would be necessary for long-term observation. In this regard, discomfort, irritation, microbial infection, and inflammatory issues that exist for general wearing of contact lenses will also occur in contact lens biosensors. 

The last but not the least are clinical perspectives. Although these concerns are rarely discussed to date, they can be key to the prevalence of the contact lens biosensors. An important aspect of which is the management of continuous monitoring of diseases and drug administration. The well coupling between both actions is critical to some diseases, such as diabetes. Another consideration is comfort of wear. This demand becomes more difficult to achieve after integrating too many components with the contact lens platform. Nevertheless, this factor will determine the actual length of time to be accepted by wearers. In addition, an underlying concern is the potential high cost due to the sophisticated fabrication of the device. This concern may form a barrier to prevent the contact lens sensors from being disposable. Clinical acceptance can also be an issue since data measured from tear is non-standard as compared to blood. How to bridge the gap between blood and other body fluids and convince clinicians their feasibility needs time and tremendous effort.

### 4.2. Suggestions

The easiest method to overcome one of the major disadvantages of enzymatic electrochemical sensors is to remove the dependence on enzymatic reaction. Non-enzymatic sensors, which employ metal surfaces, such as platinum, copper oxide or gold that replace GOD as catalysts [105,106,107,108]. However, these sensors typically have reduced sensitivity and selectivity, in comparison to enzymatic-based sensors [109]. There have been recent developments in improving the sensitivity of these non-enzymatic sensors by utilizing nanostructured surfaces to increase the reaction surface area [108,110]. However, many of these nanostructures may be difficult to reproduce and may affect the functionality of contact lenses.

**Figure 11:**
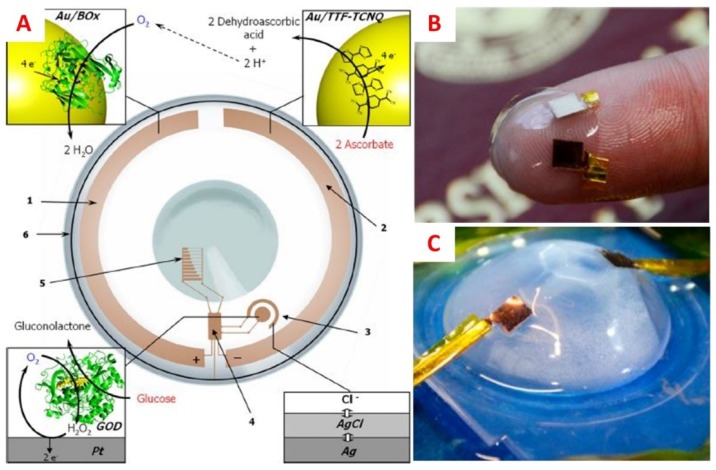
(**A**) Schematic illustration of a bionic contact lens consisting of (1) gold nanoparticle and bilirubin oxidase cathode; (2) gold nanoparticle and tetrathiafulvalene-tetracyanoquinodimethane (TTF-TCNQ) complex anode; (3) glucose sensor; (4) interface chip; (5) display; and (6) an antenna. The biofuel cell provides electrical energy to the other components by utilizing ascorbate in tear fluid. Reprinted and adapted with permission [116]. Copyright 2013, American Chemical Society. (**B**) A lactate/oxygen biofuel cell on a wearable contact lens, consisting of gold-modified electrodes immobilized with lactate oxidase on the working anode, and bilirubin oxidase on the counter/reference cathode. The biofuel cell was tested in a two-electrode system and sandwiched between two contact lenses to prevent contact with the eye. (**C**) The setup used to test the performance of the fabricated biofuel cell. Reprinted and adapted with permission [117]. Copyright 2018, American Chemical Society.

Various studies [39,85,111,112,113] have attempted to remedy the issue of supplying power to electrochemical contact lens sensors via wireless radiofrequency, induction, solar power, or integrating biofuel cells that utilize components of tear fluid to chemically generate power. However, integration with wireless power sources requires peripheral equipment, compromising the ease of use of this method. By contrast, biofuel cells can provide an alternative source of power by converting tear fluid constituents to electrical energy via low cost miniaturized circuits [114]. For many electrochemical sensors, glucose would be the first choice as a fuel source; whereas for glucose-sensing devices, ascorbate may be a more appropriate alternative [115]. Falk et al. [116] was able to demonstrate that adequate electrical power could be generated from basal tears, with no influence on the glucose concentration within the tear fluid (Figure 11A). Additionally, lactate could also serve as a potential biochemical feed for generation of electrical current, as demonstrated by the Magner group (Figure 11B) [117]. The advances in fabricating stable power sources and low-powered electronics can aid in facilitating the realization of self-powered, minimally invasive, and continuous monitoring of diseases, such as diabetes.

Although there might not be immediate solutions to all the challenges from the clinical perspectives, attempting advanced microfabrication and new materials for the contact lens biosensors is absolutely a way to reduce the cost and improve the comfort of wear. In addition, a new market is also necessary to sustain the development of new gadgets. To this end, more clinical trials should be conducted to pave a way for bringing the devices from theory to practice. Eventually, clinical acceptance relies on such big data to form substantial evidences of support.

## 5. Conclusions

The development of contact lens sensor technology has gained traction in the past decade largely due to advances in the miniaturization of electrical circuits and the identification of relevant biomarkers in the tear fluid. This sensor platform has several advantages, including minimally invasive, continuously monitoring of biomarkers. Additionally, smart contact lenses appeal more to patients because of the familiarity of the product and ease of use. However, great improvements are still under work for such types of platform, such as specificity, sensitivity, biocompatibility, integration with readout circuitry, and reproducibility to achieve practicality. With these issues resolved, it is expected that the use of contact lens biosensors will lead to better personalized medical treatment. As an emerging technology, contact lens biosensors still face challenges as they move from theory to practice. However, numerous studies up to date have demonstrated the potential of this platform. To this end, other advanced technologies and fabrication techniques have been incorporated into this platform, such as graphene coatings, nanoparticles, and quantum dots to provide readouts compatible with existing electronic devices (smartphones, laptops, and tablets) [82,102,118]. The pairing of sensors and mobile technology can facilitate real-time data acquisition and transfer to physicians for efficient diagnosis. Notably, this platform will be able to assist future drug therapies and treatments as well. Additionally, a greater understanding of the link between disease and ocular biomarker concentrations are required to enable the practicability of multifunctional contact lens biosensors. 

## Figures and Tables

**Table 1:** Tear film compositions and functions.

Tear Layer	Primary Function	Source/Composition	Ref.
Lipid (outer)	Meibomian glands—low (wax and cholesterol esters) and high (triglyceride, fatty acids, and phospholipids) polarity lipids	Enables formation of a thin tear film, stabilizes the aqueous layer by suppressing evaporation, as well as preventing microbial infection	[29]
Aqueous (middle)	Lacrimal glands—inorganic salts, enzymes, metabolites, and proteins	Provides oxygen to the corneal epithelium, lubricates the eye, washes away foreign particles and irritants, and can also protect from infection (lysozyme and β-lysine)	[28,30]
Mucin (inner)	Conjunctival goblet cells (and corneal and conjunctival epithelium)—glycoproteins	Hydrophilic interfacial layer over the ocular surface that forms a protective film over the epithelial cells	[28,30]

**Table 2:** Typical concentrations of tear fluid constituents.

Component	Concentration	Ref.
Na^+^	120–165 mM	[15,36,37]
K^+^	15–42 mM	[15,38]
Cl^−^	118–135 mM	[15,19]
Mg^2+^	0.5–1.1 mM	[15,38]
Ca^2+^	0.4–1.1 mM	[15,38]
HCO_3_^−^	20–42 mM	[15]
Urea	6 mM	[39]
Ascorbate	11–23 µM	[40,41]
Lactate	1–5 mM	[42]
Glucose *^,^**	0.1–0.6 mM	[15,39]
Total Protein	5–11 mg/mL	[15,43]

* Blood glucose concentrations for a healthy person and diabetics are 3–8 mM and 2–40 mM, respectively [44]. ** Tear glucose concentration for diabetics is 0.5–5 mM [45].

**Table 3:** Identified biomarkers in tear fluid associated with diseases and conditions. Adapted from [24,28,50,51].

Condition/Disease	Biomarkers
Allergic conjunctivitis	Ig gamma-2, leukocyte elastase inhibitor, sPLA2-IIa, total protein, serum albumin precursor
Autoimmune thyroid eye disease	interleukin-1β, IL-6, IL-7, IL-13, IL-17A, IL-18, TNF-α, RANTES/CCL5, IFN-γ
Blepharitis	Proteomics and lipodomics, serum albumin precursor, α-1 antitrypsin, lacritin precursor, lysozyme, Ig-κ chain VIII, prolactin inducible protein (PIP/GCDFP-15), cystatin-SA III, pyruvate kinase, phosphoethanolamine, sphingomyelin
Cancer	Lacryglobin, sulf-1, cystatin SA, 5-AMP-activated protein kinase subunit γ-3, triosephosphate isomerase, microtubule-associated tumor suppressor 1, keratin (type I) putative LCN-1 like protein, malate dehydrogenase, Ig α-2 chain c region, Ig heavy chain VIII region, protein S100-A4, keratin (type II), pericentrin, complement C1q subcomponent subunit C
Conjunctivochalasis	S100 (A8, A9, A4), guanosine triphosphate-binding protein 2, l-lactate dehydrogenase A-like 6B, fatty acid-binding protein, keratin type I cytoskeletal 10, gluthathione S-transferase P, peroxiredoxin-1, peroxiredoxin-5, cullin-4B+ glyceraldehydes 3-phosphate dehydrogenase, Pro-MMP-9
Cystic Fibrosis	IL-8, IFN-γ, MIP-1α, MIP-1β
Diabetic retinopathy	NGF, LCN-1, lactotransferrin, lysozyme C, lacritin, lipophilin A, Ig lambda chain, HSP27, B2M, TNF-α, N- and O-linked glycans
Dry eye	Proteins: Lysozyme, lactoferrin, LPRR4, calgranulin A/S100 A8, LPRR3, nasopharyngeal carcinoma-associated PRP4, α-1 antitrypsin α-enolase, α-1 acid glycoprotein 1, S100 A4, S100 A11 (calgizzarin), S100 A9/calgranulin B, LCN-1, mammaglobin B, lipophilin A, B2M, S100A6, annexin A1, annexin A11, CST4, PLAA, transferrin, defensin-1, clusterin, lactotransferrin, cathepsin S, anti-SS-A, anti-SS-B, anti-α-fodrin, malate dehydrogenase (MDH) 2, palate lung nasal clone (PLUNC), MUC5AC, NGF, CGRP, NPY, serotonin, IL-1, IL-2, IL-5, IL-6, IL-8/CXCL8, IL-10, IL-12, IL-16, IL-33, GCSF, MCP1/CCL2, MIP1d (CCL15), ENA-78/CXCL5, sILR1, sIL-6R, sgp, sEGFR, sTNFR, IL-17A, IL-21, IL-22, IL-1RA, CXCL9/MIG, CXCL11/I-TAC, CXCL10/IP-10, MIP-1β/CCL4, RANTES/CCL5, EGF, TNF-α, IFN-γ, MMP-9, MIP1-α/CCL3, VEGF, fractalkine, OAHFA, lysophospholipids, PUFA-containing diacylglyceride, HEL, HNE, MDA, cholesterol, N-acetylglucosamine, glutamate, creatine, amino-n-butyrate, choline, acetylchoine, arginine, phosphoethanolamine, glucose, phenylalanine
Glaucoma	Autoantibodies—HSP10, HSP27, MBP, Protein S100, BDNF, immunoglobulins, PIP, lysozyme C, LCN-1, lactotransferrin, PRP4, PIP, zinc-α2-glycoprotein, polymeric immunoglobulin receptor, cystatin S, Ig-γ chain C region, Ig-α-2 chain C region, immunoglobulin J chain, Ig α-1 chain, MUC5AC, Hcy
Herpes Simplex Virus	HSV-specific IgA and IgG antibodies
Keratoconus	Llactoferrin, IgA, GCDFP-15/PIP, RANTES/CCL5, MMP-13, NGF, IL-6, MMP-9, IL-1β, IFN-γ, SFRP-1, prolidase
Keratopathy	N-linked glycoproteins, cytokines, gelatinases, MMP-2, -9, -10, TIMP-2
Ocular allergy	Proteins: neutrophil myeloperoxidase, ECP, eosinophil, neurotoxin, sIL-2 receptor, histamine, MMP-1, MMP-9, TIMP-2, haemopexin, substance P, CGRP, VIP, transferring, mamaglobin B, secretoglobin 1D, IgE Cytokines/chemokines: IL-1α, IL-1β, IL-2, IL-6, IL-12, IL-13, eotaxin-1/CCL11, RANTES/CCL5, MCP-1/CCL2, IL-4, IL-5, IL-10, sIL-6R, eotaxin-2/CCL24, TNF-α, IFN-γ, IL-5, IL-10
Ocular chlamydia trachomatis	IgA, antichlamydial IgG
Ocular GVHD	Cytokines/chemokines: IL-6, IFN-γ, soluble TNF receptor 1 (sRNFR1), IL-2, IL-10, IL-17A, TNF-α, EGF, IL-1RA, IL-8/CXCL8, IP10/CXCL10
Ocular rosacea	Matrix metalloproteinase-8 (MMP-8), oligosaccharides
Peripheral Ulcerative Keratitis	MMP-2, MMP-9
Pterygium	α-defensins, S100A8, A9
Sjörgen’s syndrome	Proteomics, lysozyme, epidermal growth factor, AQP5, IL-1α and β, IL-6, IL-8, TGF-β1, IL-1Ra, TNF-α, MUC5AC, GalNAc transferase, GalNAc-T2, -T6 isoenzymes, O-glycan residues, MMP-9
Trachoma	Immunoglobulins, IgG against cHSP60, CPAF, CT795, EGF, TGF-β1, TNF-α
Thyroid Ophthalmopathy	IL-1β, IL-6, IL-13, IL-17A, IL-18, TNF-α, RANTES/CCL5, IL-7

**Table 4:** Comparison of different types of contact lens biosensors.

Method		Range	Readout Time	Analyte	Power	Ref.
Fluorescence		50 μM–100 mM	<45 min	glucose	No	[44,45]
Photonic Crystal	1D	0–50 mM	<30 min	glucose	No	[82]
	2D, 3D	0–50 mM	3 min	glucose	No	[83]
		0–50 mM	30 min	glucose	No	[84]
Electrochemistry		0–2 mM	<hundred seconds	glucose	Yes	[85]
		50 μM–5 mM	35 s	l-lactate	Yes	[42]
		30 μM–5 mM	<15 s	glucose	Yes	[86]
